# *Lasiodiplodia theobromae* as a Producer of Biotechnologically Relevant Enzymes

**DOI:** 10.3390/ijms19020029

**Published:** 2018-01-23

**Authors:** Carina Félix, Sofia Libório, Mariana Nunes, Rafael Félix, Ana S. Duarte, Artur Alves, Ana C. Esteves

**Affiliations:** Department of Biology, CESAM—Centre for Environmental and Marine Studies, University of Aveiro, Campus Universitário de Santiago, 3810-193 Aveiro, Portugal; carinafelix89@gmail.com (C.F.); sofia.liborio@ua.pt (S.L.); mariana.nunes@ua.pt (M.N.); rafaelfariafelix95@gmail.com (R.F.); asduarte@ua.pt (A.S.D.); artur.alves@ua.pt (A.A.)

**Keywords:** extracellular enzymatic activity, temperature, proteases, endoglucanases, lipases, thermostability

## Abstract

Phytopathogenic fungi are known to produce several types of enzymes usually involved in plant cell wall degradation and pathogenesis. The increasing of global temperature may induce fungi, such as *Lasiodiplodia theobromae* (*L. theobromae*), to alter its behavior. Nonetheless, there is only limited information regarding the effect of temperature on *L. theobromae* production of enzymes. The need for new, thermostable enzymes, that are biotechnologically relevant, led us to investigate the effect of temperature on the production of several extracellular enzymatic activities by different *L. theobromae* strains. Fungi were grown at 25 °C, 30 °C and 37 °C and the enzymatic activities were detected by plate assays, quantified by spectrophotometric methods and characterized by zymography. The thermostability (25–80 °C) of the enzymes produced was also tested. Strains CAA019, CBS339.90, LA-SOL3, LA-SV1 and LA-MA-1 produced amylases, gelatinases, caseinases, cellulases, lipases, laccases, xylanases, pectinases and pectin liases. Temperature modulated the expression of the enzymes, and this effect was more visible when fungi were grown at 37 °C than at lower temperatures. Contrary to proteolytic and endoglucanolytic activities, whose highest activities were detected when fungi were grown at 30 °C, lipolytic activity was not detected at this growth temperature. Profiles of proteases and endoglucanases of fungi grown at different temperatures were characterized by zymography. Enzymes were shown to be more thermostable when fungi were grown at 30 °C. Proteases were active up to 50 °C and endoglucanases up to 70 °C. Lipases were the least stable, with activities detected up to 45 °C. The enzymatic profiles detected for *L. theobromae* strains tested showed to be temperature and strain-dependent, making this species a good target for biotechnological applications.

## 1. Introduction

Fungal plant pathogens are known to express high amounts of hydrolytic and oxidative enzymes [1,2,3,4]. The lack of specialized host penetration structures makes hydrolytic and oxidative enzymes crucial to degrade plant cell wall and extracellular matrices, which are plants’ major barriers [5,6]. Although the composition and structure of plant cell walls differ among plant lineages, they share basic constituents, as cellulose microfibrils embedded in a matrix of pectin, hemicellulose, lignin, and structural proteins [6]. Also, fungal nutrition is achieved via the absorption of small constituents obtained by the enzymatic catabolism of environmental complex organic polymers, such as cellulose or proteins [3,4]. Thus, the most highly recognized extracellular hydrolytic enzymes involved in plant invasion include cellulases, proteases, and lipases. Cellulases are responsible for catalyzing the hydrolysis of cellulose into simple sugars, weakening the plant cell wall [7]. Proteases are known to degrade proteins of the cell wall, facilitating hyphal penetration through it, and to modify chemical signals involved in the plant defense mechanisms [4]. In contrast, lipases also contribute to the degradation of the plant cell wall, but the exact mechanism remains widely unknown [4,8]. 

*Lasiodiplodia theobromae* (Pat.) Griff. and Maubl. is a phytopathogenic fungus from the family Botryosphaeriaceae, typically found in tropical and subtropical regions [9,10]. It is widely distributed, but mostly confined to 40° North and 40° South of the equator, presenting its optimal growth temperature between 27 °C to 33 °C [11]. It has been considered a latent pathogen but *L. theobromae* is also able to establish endophytic infections [12] and was associated to approximately 500 hosts, mostly woody plants [10,13]. It has also been associated with human infections, behaving as an opportunist pathogen [14,15,16,17]. 

Filamentous fungi are the preferred source of industrial enzymes due to their easy cultivation, and high production of extracellular enzymes [18,19,20]. Several types of enzymes produced by filamentous fungi are found available in the market: glucoamylases, cellulases, lipases, pectinases, laccases, catalases, proteases, and others [18,19,20]. Among them, *Aspergillus* species dominate the production of many enzymes [21,22,23]. These enzymes are currently applied in the production of detergents, starch, drinks, food, textile, animal feed, chemicals, and biomedical products [18,19,20]. Phytopathogenic fungi, known to produce a huge arsenal of extracellular enzymes to penetrate the plant host, can be a good source of enzymes with biotechnological potential [24].

The great ability of adaptation to different environments, the capacity to infect a vast number of hosts and the expression of high amounts of extracellular enzymes makes *L. theobromae*, a potential producer of biotechnologically relevant enzymes [4,20].

The goal of this work was to characterize the extracellular enzymatic activities of different strains of *L. theobromae* and evaluate the impact that temperature has on their production and activity.

## 2. Results

### 2.1. Extracellular Enzymatic Activity

**Figure 1:**
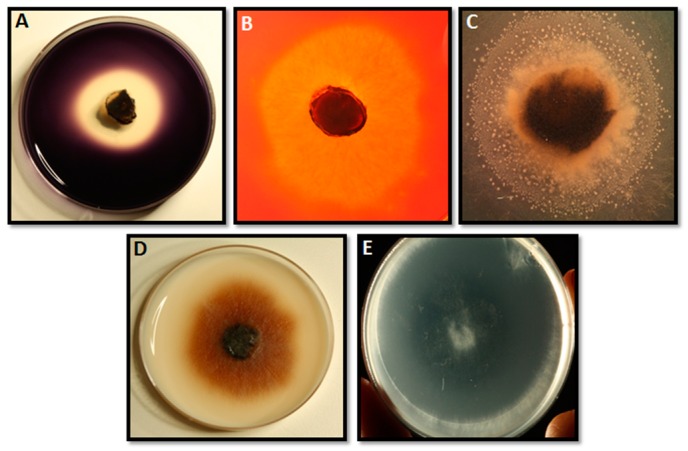
Images showing positive results of extracellular enzymatic activity of all types of enzymes tested. (**A**) amylases; (**B**) cellulases/xylanases; (**C**) lipases; (**D**) laccases; (**E**) proteases.

**Figure 2:**
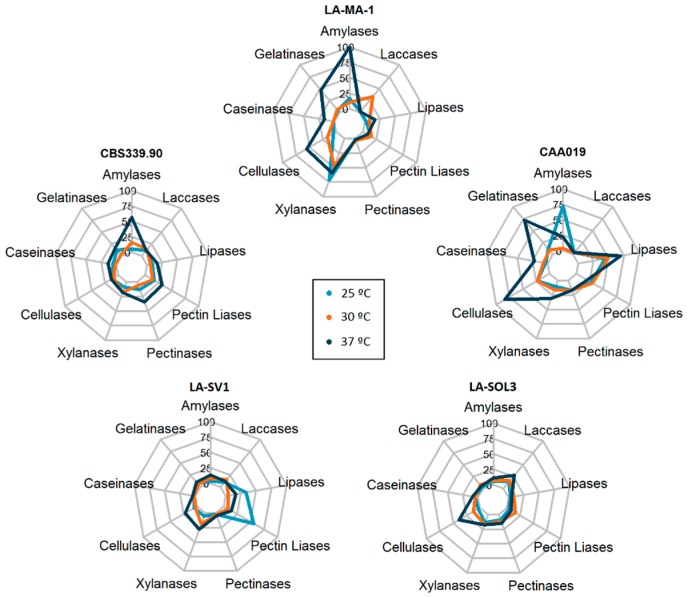
Extracellular enzymatic activity (%) of *L. theobromae* strains after a 48-h incubation period. The percentages were calculated using the higher enzymatic index determined as 100%.

Three temperatures were selected for testing: 25 °C, 30 °C and 37 °C. Those include the average temperature of tropical and subtropical regions infected by this species (25 °C), the optimal growth temperature of the fungus (30 °C) [25] and the human body temperature (37 °C). This allows us to compare the effect that optimal and stress temperatures have on the enzymatic profile of these strains. All strains secreted amylases, cellulases, xylanases, pectinases, pectin lyases, gelatinases, caseinases, lipases and laccases when grown at 25 °C, 30 °C and 37 °C (Figure 1). Figure 2 shows the enzymatic index (%) of all tested enzymes for all the strains grown at 25 °C, 30 °C and 37 °C.

It is possible to verify that, globally, the enzymatic indexes at 37 °C for all the enzymes tested were more variable than those obtained at 25 °C and 30 °C. It is important to highlight that the expression of gelatinases, cellulases and lipases, by strain CAA019 grown at 37 °C (Figure 2-CAA019) and of amylases by strain LA-MA-1 (Figure 2-LA-MA-1) was significantly higher (*p* < 0.0001) than when the strain was grown at 25 °C or 30 °C. For the clinical strain, CBS339.90 (Figure 2-CBS339.90), the profiles obtained after growth at 25 °C and 30 °C were similar, being the extracellular activity significantly higher at 37 °C (*p* < 0.0001) for most enzymes (amylases, gelatinases, caseinases, pectinases and pectin liases). For the strains isolated from grapevines, LA-SV1 and LA-SOL3, the activities were low at all growth temperatures (Figure 2-LA-SV1 and LA-SOL3).

**Figure 3:**
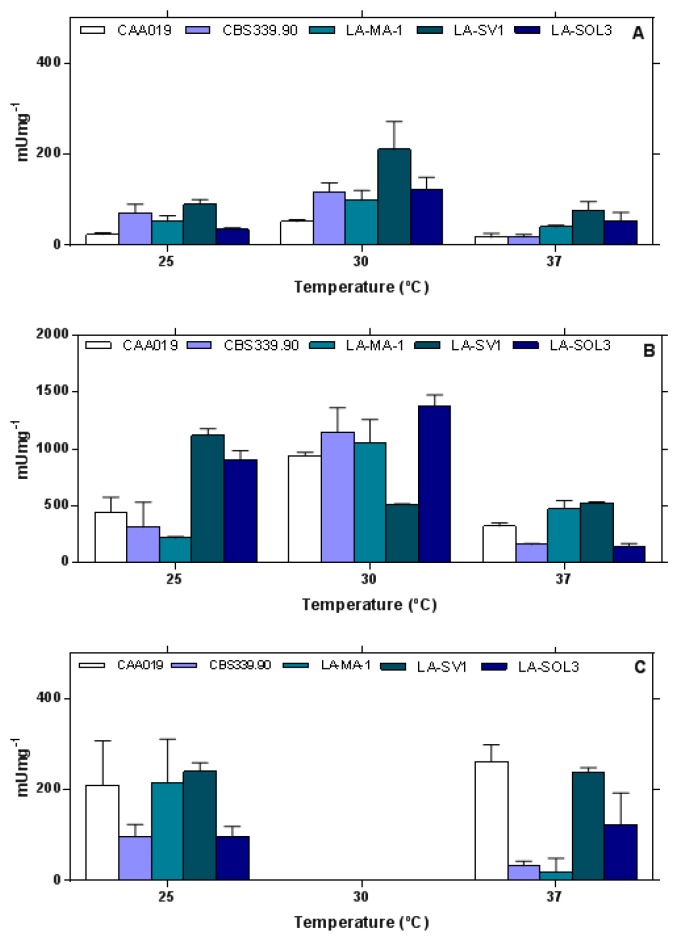
Extracellular endoglucanolytic (**A**); proteolytic (**B**) and lipolytic (**C**) activities of the 5 strains of *L. theobromae* grown at 25 °C, 30 °C and 37 °C and tested at the same temperature of growth. Data is presented as average ± standard deviation, normalized for mycelium dry weight.

Although the plate assay is very useful to evaluate the presence/absence of a specific enzyme, only a semi-quantification of the activity can be carried out. Thus, some enzymes were selected to be quantified due to their relevance for fungal pathogenicity processes as well due to their biotechnological potential: endoglucanases, proteases and lipases (Figure 3).

Globally, strains grown at 30 °C presented higher enzymatic activities than when grown at 25 °C and 37 °C. Lipolytic activity was not detected when the strains were grown at 30 °C. The expression of proteases by *L. theobromae* strains was higher than the expression of lipases and endoglucanases, especially when fungi were grown at 30 °C. Endoglucanolytic activities of strains LA-MA-1, LA-SV1 and LA-SOL3 grown at 30 °C were significantly higher than when the fungi were grown at 25 °C or 37 °C (*p* < 0.0001). Endoglucanolytic activity of strain CAA019 was independent of growth temperature.

Proteolytic activity (Figure 3B) was significantly higher when strains were grown at 30 °C (*p* < 0.001). 

The lipolytic activity detected when fungi were grown at 25 °C and 37 °C was similar for all strains (Figure 3C), except for strains CBS339.90 and LA-MA-1, where a significant lower activity was detected at 37 °C (*p* < 0.05 and *p <* 0.0001, respectively). 

**Figure 4:**
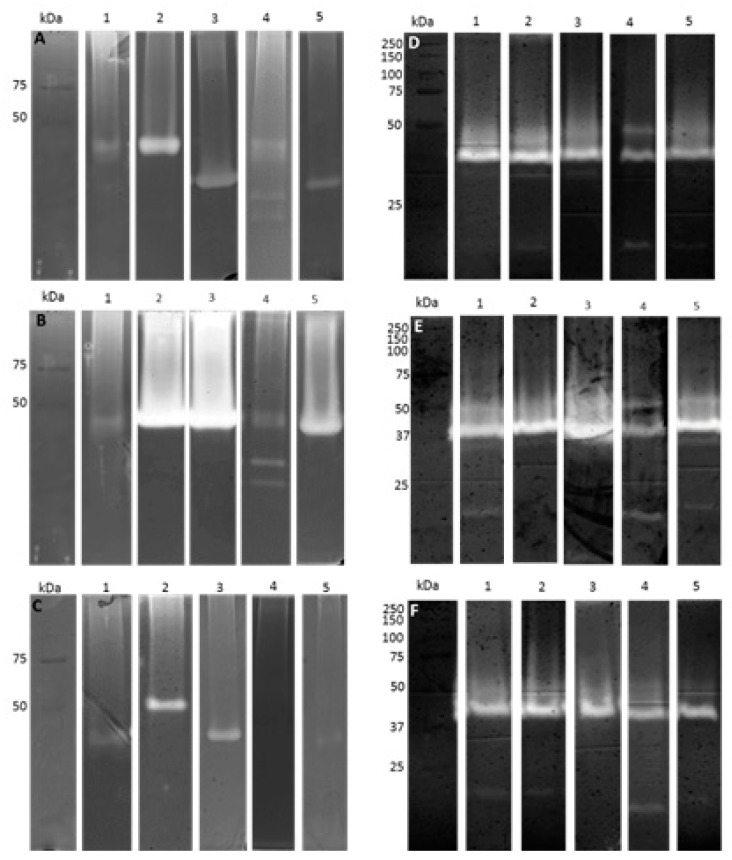
Extracellular gelatinolytic (**A**–**C**) and endoglucanolytic (**D**–**F**) activities of 5 strains of *L. theobromae* grown at 25 °C (**A**/**D**), 30 °C (**B**/**E**) and 37 °C (**C**/**F**). 1- LAMA-1, 2- CBS339.90, 3- LA-SV1, 4- CAA019, 5- LA-SOL3. Each gel is representative of three independent runs.

The crude extracts were also characterized by sodium dodecyl sulfate (SDS)-zymography analysis. Figure 4 shows the proteolytic and endoglucanolytic profiles for all strains grown at 25 °C, 30 °C and 37 °C. Lipolytic activities were not detected by zymography, either because activity is below the limits of detection of the technique, or because these enzymes were not able to digest olive oil.

Proteolytic profiles varied depending on the temperature of growth and on the strain. Enzymes with apparent molecular weights between 45.5 and 20.9 kDa (Figure 4A–C) were detected. The most intense bands were detected for strains CBS339.90, LA-SV1 and LA-SOL3, grown at 30 °C. Cellulolytic profiles were similar between strains and growth temperatures: it was possible to detect bands with apparent molecular weights between 51.3 and 11.5 kDa (Figure 4D–F). However, at 30 °C, the bands are more intense when compared with the profiles obtained for 25 °C and 37 °C. This agrees with activity quantification, where the growth temperature that led to higher activities was 30 °C. At 37 °C, the clinical strain secreted less proteases than some environmental strains (CAA019, LA-MA-1 and LA-SV1). However, at 37 °C, strain CBS339.90 exhibited a different and more intense profile than the other strains.

### 2.2. Thermostability of Endoglucanases

**Figure 5:**
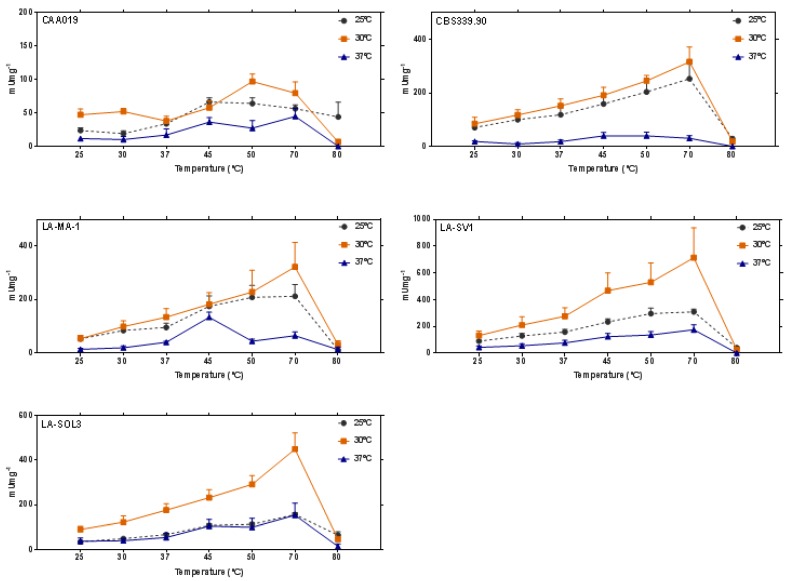
Extracellular endoglucanolytic activity of strains CAA019, CBS339.90, LA-MA-1, LA-SV1 and LA-SOL3 grown at 25 °C, 30 °C and 37 °C and assayed at 7 temperatures. Data is presented as average ± standard deviation.

Endoglucanolytic activity was quantified for all strains grown at 25 °C, 30 °C and 37 °C (Figure 5) at increasing reaction temperatures (25–80 °C). There is a general increase of activity from 25 °C to 70 °C, and a strong decrease at 80 °C.

### 2.3. Thermostability of Proteases

**Figure 6:**
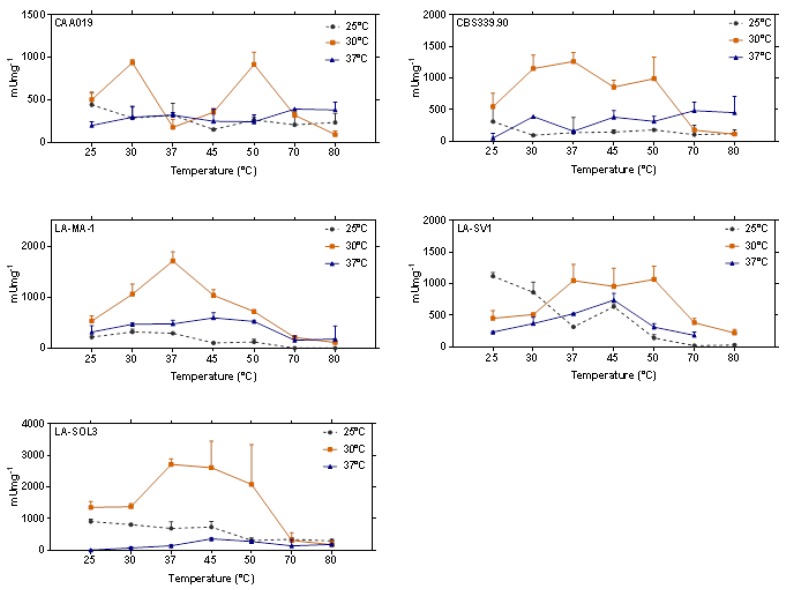
Extracellular proteolytic activity of strains CAA019, CBS339.90, LA-MA-1, LA-SV1 and LA-SOL3 grown at 25 °C, 30 °C and 37 °C and tested at 7 different temperatures. Data is presented as average ± standard deviation.

At 30 °C growth temperature, there is a general increase of activity until 50 °C and a decrease at 70 °C and 80 °C. At growth temperatures of 25 °C and 37 °C the pattern of proteolytic activity is consistent with the presence of several enzymes, with different optimal temperatures (Figure 6).

### 2.4. Thermostability of Lipases

**Figure 7:**
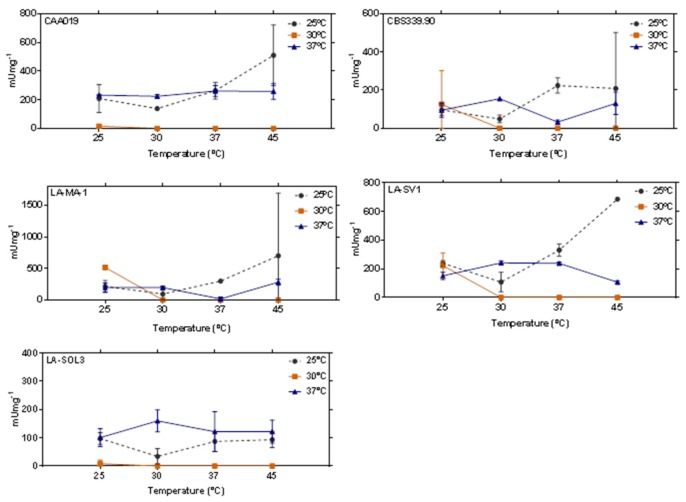
Extracellular lipolytic activity of CAA019, CBS339.90, LA-MA-1, LA-SV1 and LA-SOL3 grown at 25 °C, 30 °C and 37 °C and tested at 4 different temperatures. Data is presented as average ± standard deviation.

Lipolytic activity was tested for all strains grown at 25 °C, 30 °C and 37 °C. However, due to the spontaneous degradation of the substrate at high temperatures, only 4 different temperatures of incubation were tested (25 °C, 30 °C, 37 °C and 45 °C), (Figure 7). For strains grown at 25 °C and 37 °C, extracellular lipolytic activity was detected for all temperatures tested. However, when strains were grown at 30 °C it was detected only at 25 °C, being absent at 30 °C, 37 °C and 45 °C incubation. For extracellular lipases, the global average remained below 500 mU mg^−1^, with no significant trend with increasing temperature.

### 2.5. Multi Factor Analysis of Extracellular Activity

**Figure 8:**
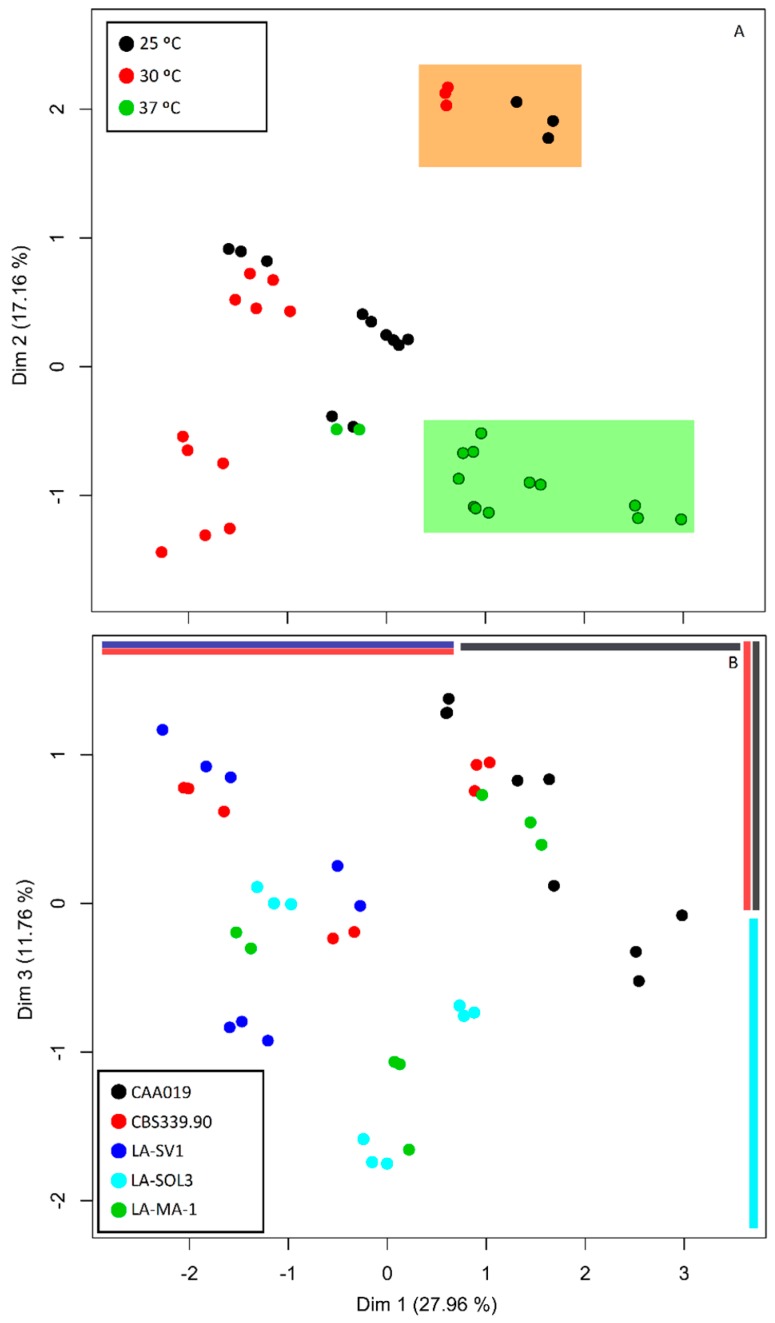
Multi factor analysis of detection, quantification and characterization of extracellular enzymatic activity data at 25 °C, 30 °C and 37 °C *L. theobromae* strains using the growth temperature (**A**) and the strains (**B**) as factors. Post-hoc analysis of the scores of both dimensions 1 and 3 revealed the one-dimensional separations (colored bars: top–horizontal, right–vertical).

A multifactor analysis (MFA) MFA was conducted in order to be able to infer which of the factors—temperature or strain—had the major influence in the expression of the enzymes in study (Figure 8).

Dimensions 1 and 2 (Figure 8) account to 43.9% of the variance of the data, providing a good reduction of dimensions in our data. Dimension 1 summarizes a total of 27.3% of the variance, of which 77.2% comes from enzymatic detection and quantification data. Dimension 2 accounts for 16.6% of the variance, mainly explained by zymography data (70.9%) and dimension 3 provides a scoring that despite its lower variance (11.8%) has the advantage of resolving quantity and identity information of enzymes in equal proportions.

Using the growth temperature as a factor (Figure 8A), dimensions 1 and 2 were shown to effectively produce different scores for our samples (*p* < 0.001 for Kruskal–Wallis test). It is possible to verify that one cluster is formed by data from fungi grown at 37 °C (green-shaded box in Figure 8A), validated by a post hoc Nemenyi analysis. At dimension 1, *L. theobromae* strains grown at 37 °C have significantly higher scores than those grown at 30 °C (*p* < 0.001). At dimension 2, scores were significantly lower for data from fungi grown at 37 °C than at 30 °C or 25 °C (*p* < 0.05 and *p* < 0.001, respectively). 

Strain was also analyzed as a factor in the MFA (Figure 8B). Dimension 2 did not significantly separate strains (*p* > 0.05). Thus, dimension 3 (*p* < 0.01) was plotted and analyzed with dimension 1 (*p* < 0.001) as a function of strains. Grouping by strains is not clear upon visual inspection of the MFA. However, post-hoc analysis of the scores of both dimensions 1 and 3 revealed the one-dimensional separations depicted in Figure 8B (colored bars: top–horizontal, right–vertical). In dimension 1, a significant difference between strain CAA019 and both strains CBS339.90 (*p* < 0.05) and LA-SV1 (*p* < 0.001) was found, suggesting the peculiarity of strain CAA019 concerning extracellular enzymatic activity. In dimension 3, which has equal contributions of enzymatic activities and molecular weight-based identities of enzymes, strain LA-SOL3 was distinguished from strains CAA019 (*p* < 0.05) and CBS339.90 (*p* < 0.05).

## 3. Discussion

### 3.1. Extracellular Enzymatic Activity

There are indications that plant pathogens may secrete different quantities of host-specific cell wall degrading enzymes (CWDEs) [26,27]. Thus, if a pathogen infects plants, it is expected that the enzymes secreted will be related to specific substrates such as, cellulose, hemicellulose and lignin [5]. The same happens with other hosts: if a pathogen infects animals, a secretion of enzymes able to degrade mammalian-specific substrates is expected. Therefore, it was surprising to verify that the cellulolytic activity, among the strains of *L. theobromae* studied was low. However, only endoglucanases were evaluated, and the hydrolysis of insoluble cellulose requires multiple cellulases (endoglucanases, exoglucanases and β-glucosidases) acting simultaneously to convert the complex matrix into simple sugars [7]. 

Esteves and colleagues [4] found that several species of *Lasiodiplodia* express active endoglucanases active at high temperatures (50 °C). Our data agrees with that previous report, since we showed an increase of the endoglucanolytic activity up to 70 °C and a marked decrease only at 80 °C [4]. 

The absence of lipolytic activity at the optimal fungal growth temperature, 30 °C, was not expected (Figure 3), suggesting that this class of hydrolytic enzymes is associated with adaptive responses to temperature. The expression of lipases only at 25 °C and 37 °C, at which pathogenesis has been reported (see [25,28]), might implicate a role of lipases in the virulence of *L. theobromae*. The importance of lipolytic activity in opportunistic fungal human infections has long been discussed [8,29]. While a role of hydrolytic enzymes in host penetration has been generally recognized, a more intricate mechanism of lipase-induced immune modulation was characterized in *Candida parapsilosis* [30], providing a stronger link of these enzymes to this organism’s pathogenicity. Also, the existence of a waxy cuticle in the leaves, young shoots and other aerial plants has been similarly proposed to be the reason for the lipase-associated virulence [31], through tissue invasion. Although some examples of lipase knock-outs with retained virulence have been published [32], definite evidence for a lipase-mediated pathogenicity mechanism was obtained by Voigt et al. [33], which managed to drastically reduce symptoms in *F. graminearum* infected plants using a potent lipase inhibitor, Ebelactone B [33]. Alternatively, it may reveal that these fungi do not rely on fatty acids and glycerol (the products of triacylglycerols’ digestion) for nutrition at their optimal growth temperature. 

### 3.2. Multi Factor Analysis of Extracellular Activity

In MFA, a principal component analysis (PCA) is performed on each quantitative data set and a multi correspondence analysis (MCA) on each qualitative data set, and the first eigenvalue of each eigenvector is used to standardize the data, which can then be merged to form a matrix on which a PCA is performed [34].

Considering the relative contributions of the different data (Figure 8A) the differences suggest that the enzymatic activity and the zymographic profiles of the strains tend to be more severely altered and more strain-independent at 37 °C. At 25 °C and 30 °C there is not a clear distinction of groups by temperature. This does not mean that those temperatures do not induce changes, but rather suggests that the changes induced are differentially determined by other factors, such as the strain. For instance, a discrete group (orange-shaded box in Figure 8A) of 25 °C and 30 °C-grown CAA019 is formed by the first two dimensions. This may mean that enzymes produced by this strain, grown at those temperatures, still constitute much more similar profiles than when compared with other strains.

When analyzing strain as a factor, the variance in data accounts for circa 39%, may indicate that different strains may have evolved by producing different enzymes, which could be an adaptation to environment (i.e., their host).

### 3.3. Thermostability

The enzymatic hydrolysis of cellulosic biomass is an alternative for the generation of sugars, that are the base materials to produce products that are commercially important, such as organic acids, bioethanol, free sugars, animal feeds and antibiotics. Since enzymes are non-polluting, specific, recoverable and do not require high levels of energy to work, they are more efficient and eco-friendly than acid or alkali hydrolysis [7]. 

Our data shows that *L. theobromae* strains express thermostable endoglucanases (active up to 70 °C), especially when the fungi are grown at 30 °C (Figure 5).

Proteases are known to have distinct functions in fungal biology, such as in nutrition and in virulence mechanisms. In industry, these proteases also have a high number of applications, such as in detergents, food industry and pharmaceutical industry or in bioremediation processes [35,36]. The higher levels of proteolytic activity found for some of the strains under study, especially when grown at 30 °C, as well as their thermostability up to 80 °C (Figure 6), and the different profiles found by SDS-Zymography (Figure 4), demonstrates the biotechnological potential of this species.

Lipases catalyze the hydrolysis of triacylglycerols with long fatty acid chains in free fatty acids and glycerol, which makes these enzymes essential to several biotechnology industries, such as the pharmaceutical industry, detergent and soap industries and cosmetics industry, among others [37,38]. These enzymes are considered as one of the leader biocatalysts, contributing to a capital underexploited of bioindustry [37]. 

Amongst microorganisms, filamentous fungi are important producers of lipases, mostly by submerged fermentation processes [19]. This kind of process to produce lipases contributes to high levels of activity with low cost and residue formation for the industry [37]. Our results showed that all strains tested presented lipolytic activity up to 45 °C (Figure 7), showing a good potential for a future application in several industries that deal with high-temperature processes [39]. The major advantages of catalysis at higher temperatures include the high rate of product formation, high dissolution of hydrophobic substrates, high conversion efficiency and a low chance of contamination [39].

## 4. Materials and Methods

### 4.1. Microorganisms

The strains used in this study were*: L. theobromae* strains LA-SOL3 and LA-SV1, isolated from *Vitis vinifera* in Peru [13], strain CAA019, isolated from *Cocos nucifera* L. in Brazil, strain LA-MA-1, isolated from *Mangifera indica*, in Peru and strain CBS339.90, isolated from a phaeohyphomycotic cyst of a patient from Jamaica. Strain CBS339.90 was obtained from the Westerdijk Fungal Biodiversity Institute. Cultures were maintained on Potato Dextrose Agar medium (PDA, Merck, Darmstadt, Germany). For liquid growth, two plugs (5 mm diameter) from a culture with 4 days were inoculated into a 250 mL flask containing 50 mL of the desired medium and incubated at 25 °C, 30 °C or 37 °C. All assays were performed in triplicate. Culture supernatants were collected by gravitational filtration through filter paper and stored at −80 °C until use. Mycelia were dried at 50 °C for 48 h, to determine the dry weight.

### 4.2. Detection of Extracellular Enzymes

Different solid media were inoculated with a 5 mm-diameter agar plug from an actively growing culture and incubated at 25 °C, 30 °C and 37 °C, for 48 h. Data is presented as enzymatic index: the ratio between the halo of enzymatic degradation and the radial growth of the mycelium. The obtained ratio was then transformed into percentage, being the highest value of enzymatic index found used as 100%.

#### 4.2.1. Proteases

A supplement of 1% of skim milk (*w*/*v*) or gelatin (*w*/*v*) was added to the malt extract agar medium (MEA) [4]. After 48 h, the activity was detected as a clear halo around the mycelium.

#### 4.2.2. Xylanases and Cellulases

Cellulolytic and xylanolytic activity were detected by adding 1.5% agar (*w*/*v*) and 0.5% carboximethylcellulose (*w*/*v*) (CMC) for cellulases’ detection, or 0.5% xylan (*w*/*v*) and 0.1% yeast extract (*w*/*v*) for xylanase’s detection [40] to a minimal medium (MM) [0.3% NaNO3 (*w*/*v*), 0.1% KH2PO4 (*w*/*v*), 0.05% MgSO4 (*w*/*v*)]. After 48 h, plates were covered with Congo Red (Sigma, St. Louis, MO, USA) (1 mg mL^−1^) during 15 min and destained with 1 M NaCl. Activity was detected as a yellow halo around the mycelium in a red background.

#### 4.2.3. Lipases

Lipolytic activity was detected using a solid medium consisting in 1.5% agar (*w*/*v*), 1% peptone (*w*/*v*), 0.5% NaCl (*w*/*v*), 0.01% CaCl_2_ (*w*/*v*) and 1% Tween 20 (*v*/*v*) [41]. After 48 h, activity was detected as a precipitate around and under the mycelium.

#### 4.2.4. Amylases

Amylolytic activity was detected using starch agar [1% peptone (*w*/*v*), 0.5% yeast extract (*w*/*v*), 0.5% NaCl (*w*/*v*) and 0.2% starch (*w*/*v*)]. After 48 h, the plates were covered with lugol solution during 10 min and the activity was detected as a clear halo in a dark background [41].

#### 4.2.5. Pectinases and Pectin Lyases

A minimal medium supplemented with 0.1% yeast extract (*w*/*v*), 0.5% pectin (*w*/*v*) and 1.5% agar (*w*/*v*), at pH 5.0 was used to detect pectinases and the same medium at pH 7.0 for pectin lyases. After 48 h, plates were covered with 1% of cetyltrimethyl ammonium bromide. Activity was detected as a transparent halo around the mycelium [40,41].

#### 4.2.6. Laccases

For detection of laccase activity, a solid medium containing a solution of 1% of tannic acid previously sterilized was added to 1.5% (*w*/*v*) malt extract and 2% agar (*w*/*v*) [42]. After 48 h, activity was detected as an alteration of the medium color to brown around the mycelium.

### 4.3. Quantification of Extracellular Activities

#### 4.3.1. Cellulolytic Activity Quantification

A crude enzymatic extract was obtained after inoculation of two plugs with 5 mm of diameter in 50 mL of MM supplemented with 0.5% (*w*/*v*) CMC and 0.1% yeast extract (*w*/*v*). Endoglucanase activity was measured by CMC degradation, using 3,5-dinitrosalicylic acid (DNS) method to the reduction of sugars [43]. Briefly, 225 µL of enzymatic extract was added to the mixture of 75 µL of 0.1 M sodium acetate buffer, pH 5, and 1200 µL of 5% CMC, previously incubated at the desired temperature. An aliquot of 125 µL was taken at 0 min, 20 min, 60 min and 120 min of incubation and added to 500 µL of DNS, at 100 °C, during 5 min. After cooling, 750 µL of ultrapure water was added to all the tubes and the absorbance was measured at 540 nm. The blank sample was treated as all the samples, replacing the enzymatic crude by ultrapure water. As standard curve, several concentrations of glucose were prepared.

The quantification of the cellulolytic activity was made for 7 different temperatures: 25 °C, 30 °C, 37 °C, 45 °C, 50 °C, 70 °C and 80 °C. Data is presented as mean of three replicates, normalized for mycelium dry weight. 

#### 4.3.2. Proteolytic Activity Quantification

Proteolytic activity was determined as described by Macchione et al. [44], with slight modifications. A crude enzymatic extract was obtained after inoculation of two plugs (5 mm of diameter) in 50 mL of Potato Dextrose Broth (PDB) medium. An aliquot of 300 μL of crude extract was added to 1.7 mL of casein [2% (*w*/*v*), in 100 mM sodium phosphate buffer, pH 6.5] at the desired temperature and 400 μL of this mixture was added to 900 μL of 6% tricholoroacetic acid TCA (*w*/*v*) after 0 min, 5 min, 10 min and 20 min of incubation. All the samples were maintained in ice and centrifuged at 13,000 rpm for 10 min. The blank sample was prepared by replacing the enzymatic crude extract by ultrapure water and treated as all the samples, replacing the enzymatic crude extract by ultrapure water. The supernatant was used to measure the absorbance at 280 nm.

The quantification of the proteolytic activity was made at: 25 °C, 30 °C, 37 °C, 45 °C, 50 °C, 70 °C and 80 °C. Data is presented as mean of three replicates, normalized for mycelium dry weight. 

#### 4.3.3. Lipolytic Activity Quantification

Lipolytic activity was determined according to Kotogán et al. [45], with slight modifications. A crude enzymatic extract was obtained after inoculation of two plugs (5 mm of diameter) in 50 mL of PDB medium. *p*-nitrophenyl palmitate (*p*NPP; Sigma-Aldrich, Lisbon, Portugal) was used as substrate. A stock solution (3 mM) was prepared in 25% dimethyl sulfoxide and 0.5% of Triton X-100 and completed with potassium phosphate buffer (pH 6.8). A volume of 50 µL of buffered *p*NPP solution was added to 150 µL of crude extract, and incubated at the test temperature for 0 min, 45 min and 90 min. The reaction was stopped by adding 50 µL of 0.1 M sodium carbonate. The released *p*-nitrophenol (*p*NP) was measured at 405 nm in 96-well plates using a microplate reader (Biotek, Synergy HT, Winooski, VE, USA). The blank sample was treated as all the samples, replacing the enzymatic crude extract by ultrapure water. The molar absorption coefficient of *p*NP *(ε* = 1.2475 × 10^4^ M^−1^·cm^−1^) was estimated from the absorbance of *p*NP standard solutions measured at 405 nm. One enzymatic unit was defined as the amount of the enzyme that releases 1 mmol of *p*NP per minute.

The quantification of the lipolytic activity was made at 25 °C, 30 °C, 37 °C, 45 °C since higher temperatures lead to spontaneous substrate degradation. Data is presented as mean of three replicates, normalized for mycelium dry weight. 

### 4.4. Zymography

For each strain, two 5 mm-diameter plugs from an actively growing culture were inoculated on 50 mL PDB for 4 days. The enzymatic crude extract was obtained by filtration as described earlier [25].

The characterization of the extracellular enzymes was carried out by zymography [4]. Enzymatic crude extracts were diluted in sample buffer [2:1 (*v*/*v*); 62.5 mM Tris (*w*/*v*), pH 6.8, 10% SDS (*w*/*v*) and 20% glycerol (*v*/*v*)] and incubated at room temperature for 10 min. The separation of the proteins was carried out in lab casted gels (10% polyacrylamide with the correspondent substrate) in a Mini-PROTEAN 3 (Bio-Rad, Hercules, CA, USA) [46]. Electrophoresis proceeded at 100 volts for 100 min. Gels were then washed twice with 0.25% Triton X-100 (*v*/*v*) for 30 min. Gel analysis was performed after adequate staining of the gels (described below). Images were acquired on a GS-800 Calibrated Densitometer (Bio-Rad). Quantity One v. 4.6.9 (Bio-Rad) was used to estimate the apparent molecular weight (MW) of the proteins using a MW calibration kit as marker (Precision Plus Protein Standard, Bio-Rad). 

#### 4.4.1. Cellulases

Cellulolytic activity was assessed by zymography, as described previously [47] with slight modifications. The substrate CMC, 1% (*w*/*v*), was included on the preparation of the gel. After electrophoresis, the gel was incubated overnight at 25 °C in 0.05 M Tris-HCl, pH 5.0. After this period, gels were stained in Congo Red (1%), 10 min, and destained with 1 M NaCl. Enzymes with cellulolytic activity were detected as clear bands against a red background of non-degraded substrate.

#### 4.4.2. Proteases

Gelatinolytic activity was assessed by zymography, as described previously [48], with slight modifications. Gelatin [1% (*w*/*v*)] was used. After electrophoresis, the gel was incubated for 1 h at 37 °C, in 1.5 mM Tris, pH 8.8, 1 M NaCl, 1 M CaCl_2_, 2 mM ZnCl_2_, pH 7.4. Gels were stained with Coomassie Brilliant Blue R-250 [(in 50% ethanol (*v*/*v*), 10% acetic acid (*v*/*v*)] and destained with 25% ethanol (*v*/*v*) and 5% acetic acid (*v*/*v*). Enzymes with gelatinolytic activity were detected as clear bands against a blue background of non-degraded substrate.

#### 4.4.3. Lipases

Lipolytic activity was accessed by zymography as described before, [47,49] with slight modifications. A solid medium containing 0.001% of rhodamine B (*w*/*v*), 3% of olive oil (*v*/*v*) and 2.5% agar (*w*/*v*) was prepared in Tris-HCl buffer, pH 8.0 and heated with agitation until dissolution of the agar. After electrophoresis, the gel was overlaid onto the solid medium of rhodamine B/olive oil for 3 h at 37 °C. Lipolytic activity was detected exposing the plate with the gel to 365 nm, where the enzymes with activity appeared as fluorescent bands.

### 4.5. Statistical Analysis

Two-way analysis of variance (ANOVA) was used to examine the extracellular enzymatic activity profiles at different temperatures for each strain (*p* < 0.05). A Tukey’s multiple comparison test was employed to identify significant differences between the enzymatic profile of a strain grown at different temperatures. All the analyses were performed with GraphPad Prism v.7 (GraphPad Software, Inc., La Jolla, CA, USA).

A Multi Factor Analysis (MFA) was carried out to infer which factors, temperature or strain, is the most relevant on the modulation of the extracellular enzymatic profiles: detection and quantification of enzymatic activity data and zymography data (molecular weight of the bands detected). Due to the different type of data (quantitative—detection and quantification of enzymatic activity—and qualitative—presence/absence of bands in zymography and their molecular weights), MFA was considered the most suitable dimension-reducing method. The MFA was conducted with R Statistical Software [50], using the function MFAmix of the package PCAmixdata [51]. The significance of the samples groups along MFA’s first three dimensions was assessed by Kruskal-Wallis non-parametric analysis of variance of the samples’ scores by factor. The significance of pairwise differences was computed by Tukey–Kramer (Nemenyi) post-hoc test, both functions of the PMCMR package [52].

## 5. Conclusions

We showed that temperature modulates the extracellular enzymatic profile of the strains of *L. theobromae*. All strains produce an arsenal of enzymes typically involved in the degradation of plant cell walls (amylases, cellulases, xylanases, laccases, gelatinases, caseinases, lipases, pectinases and pectin liases), whose expression was influenced by the temperature of growth. These alterations show the capacity of adaption of these strains to different environments, which could also result in changes in pathogenicity. The analysis of the enzymatic profile of *L. theobromae* showed that different strains produce significantly different enzymatic profiles, indicating a strain-dependency.

The wide range of temperatures tested (from 25 °C to 80 °C) showed that these strains express thermostable enzymes (proteases, endoglucanases and lipases), which could be very interesting to several types of industries using these kinds of enzymes, such as detergents, starch, food or biomedical industries.
